# Parents’ self-directed practices towards the use of antibiotics for upper respiratory tract infections in Makkah, Saudi Arabia

**DOI:** 10.1186/s12887-019-1391-0

**Published:** 2019-02-04

**Authors:** Hani Saleh Faidah, Abdul Haseeb, Majd Yousuf Lamfon, Malak Mohammad Almatrafi, Imtinan Abdullah Almasoudi, Ejaz Cheema, Waleed Hassan Almalki, Mahmoud E Elrggal, Mahmoud M.A. Mohamed, Fahad Saleem, Manal Mansour Al-Gethamy, Beenish Pervaiz, Tahir Mehmood Khan, Mohamed Azmi Hassali

**Affiliations:** 1 Department of Medical Microbiology, Al-Noor Specialist Hospital, Ministry of Health, Makkah, Kingdom of Saudi Arabia; 2 Department of Microbiology, Faculty of Medicine, Umm Al Qura University, Makkah, Kingdom of Saudi Arabia; 3 Department of Clinical Pharmacy, College of Pharmacy, Umm Al-Qura University, Makkah, Kingdom of Saudi Arabia; 4 Department of Social and Administrative Pharmacy, School of Pharmaceutical Sciences, Universiti Sains Malaysia, Penang, Malaysia; 5 Dan Al-Majd Pharmacy, Makkah, Kingdom of Saudi Arabia; 6 Department of Pharmacology, College of Pharmacy, Umm Al Qura University, Makkah, Kingdom of Saudi Arabia; 7 Berlin-Brandenburg Center for Regenerative Therapies (BCRT) , Charite-Universitatsmedizin Berlin, Berlin, Germany; 8 Faculty of Pharmacy & Health Sciences, University of Baluchistan, Quetta, Pakistan; 9 Adult Infectious Disease Consultant and Infection Prevention and Control Programme Director, Al Noor Specialist Hospital, Makkah, Kingdom of Saudi Arabia; 10 Lady Reading Hospital, Medical Teaching Institute, Peshawar, Pakistan; 11 School of pharmacy, Monash University Malaysia, Selangor, Malaysia; 12 Institute of Pharmaceutical Sciences, University of Veterinary and Animal Sciences, Lahore, Pakistan; 13 Institute of Clinical Sciences, University of Birmingham, Birmingham, England

**Keywords:** Antimicrobial use, Upper respiratory tract infections, Parents believes

## Abstract

**Background:**

Excessive and inappropriate antimicrobial use in the community is one risk factor that can result in the spread of antimicrobial resistance. Upper respiratory tract infections are most frequently reported among children and mainly of viral origin and do not require antibiotics.

We have conducted Knowledge, Attitude and Perception (KAP) survey of parents to explore the parent’s knowledge, attitude & perception of Saudi parents.

**Methods:**

A knowledge attitude perception questioner was adopted from a previous study conducted in Greece by Panagakou et al. Raosoft online sample size calculator calculated the sample size by adding the total estimated Makkah population of 5,979,719 with a response rate of 30%, 5% margin of error and 99% confidence interval. Based on the described criteria five hundred & fifty-eight was the required sample size of the study. Incomplete questioners were excluded from the statistical analysis. SPSS version 21 was used to analyse data and to produce descriptive statistics.

**Results:**

Most of the mothers (95%) responded among parents. 67% had no health insurance to cover medications costs. Most of them (74%) were related to medium income level. Seventy per cent of the parents believed physicians as a source of information for judicious antibiotics use. Interestingly, only 8% were agreed that most of the upper respiratory tract infections are caused by viral reasons.

Majority of Saudi parents (53%) expect pediatricians to prescribe antimicrobials for their children for symptoms like a cough, nose discharge, sore throat and fever.

Moreover, most the parents had the poor knowledge to differentiate commonly used OTC medications for URTI and antibiotics like Augmentin (Co-amoxiclav), Ceclor (cefaclor) and Erythrocin (Erythromycin). While comparing males and female’s knowledge level, few males have identified Amoxil (Amoxicillin). Similarly, parents of age 20–30 years have good knowledge about the antibiotics.

**Conclusions:**

Majority of Saudi parents believe in pediatricians and use antibiotics on physician’s advice. Most of them expect antibiotics from their physicians as a primary treatment for upper respiratory tract infections. There is need for more educational activities to parents by the pharmacists to prevent antibiotics overuse among children.

## Introduction

Upper respiratory tract infections (URTIs) are common in children, and most of these are viral in origin and are often self-limiting [1–5]. Despite their viral origin, it is common practice to manage these infections with antibiotics [6]. A prospective study conducted in 13 countries suggested that even URTIs with bacterial origin can be resolved without administering antibiotics [7]. Antibiotics have limited efficacy in treating URTIs in both children as well as adults [2]. Inappropriate prescribing of antibiotics is a common practice in children [1, 4, 8] and is one of the major contributors to the emerging risk of antibiotic resistance worldwide [9–11].

Recently a study by Zhang et al. reported antibiotics use at health facilities at country, township and village level in China. They concluded the highest level of antibiotics uses among children complaining URIs, especially when visiting county hospitals [12]. Similarly, in a nationwide study by Yoshida et al., in Japan recently found that 66.4% of the preschool children attending outpatient clinics for URIs were received antibiotics and interestingly most commonly prescribed antibiotics were third-generation cephalosporin (38.3%) followed by macrolides (25.8%) and penicillin (16.0%) respectively [13].

It was described in a multicenter study conducted in eight countries that repeated antibiotic exposure was common early in life and antibiotics used for respiratory illnesses was not according to international guidelines. Among the study cohort, 39.5% of the antibiotics use was for upper respiratory tract infections. Interestingly, the highest antibiotics use was reported in South Asian countries [14].

Other factors that may contribute to the development of antibiotic resistance in children include both the attitude and practices of pediatricians [15, 16] as well as parents [17, 18]. Pediatricians often prescribe antibiotics because of parental pressure and expectation [8, 19, 20]. When parents panic due to acute illnesses in their children, they visit their pediatricians with an expectation of getting a prescription for antibiotics [21], which leads to unnecessary antibiotic use.

Parents perception towards antibiotics use is an essential factor while requesting antibiotics for their children. It has been proved by many findings that the majority of the parents believed that antibiotics are helpful to treat common cold among children and recover such symptoms promptly. This factor is more prominent in parents with poor knowledge level and lower educational level [22, 23]. In a recent systematic review regarding parenteral knowledge about antibiotics for URTIs, it is concluded that parent’s knowledge is the key factor while using antibiotics to cure their child. However, parents can be satisfied if the appropriate clarification and therapeutics plan is provided by the physicians [24].

Addressing the situation in Saudi Arabia, antibiotics are commonly prescribed to children for URTIs [25]. Furthermore, antibiotics are readily available over the counter without a prescription in Saudi Arabia [5, 26]. Evidence suggests that in 17.0% of the URTIs, parents use antibiotics while self-treating their children [26]. However, there is limited information regarding the consumption of antibiotics in the treatment of URTIs in children in the Makkah region of Saudi Arabia. There is also a need to assess parents’ knowledge, attitudes and perception towards antibiotic use in their children. This study, therefore, aims to analyse parental knowledge, behaviour and perception towards the antibiotic consumption in the treatment of URTIs in children.

### Methods

### Study design

This cross-sectional study was conducted from 1st September till 31st December 2015, using a 23- item self-administered questionnaire.

### Survey development

We followed questioner from a study conducted by SG Panagakou et al. in Greece [19]. A minor to moderate modification was done based on the Saudi context, and the final version was translated into Arabic using forward and backward translation. Besides, the face validity of the tool was assessed by conducting a pilot study among *n* = 15 respondents. The reliability of the instrument was assessed using Cronbach’s alpha value which was 0.77 for this questionnaire.

Most of the questions in the survey were closed-ended with few open-ended choices. The questioner was formatted into three main sections. Part 1 explored the demographic characteristics of the respondents. It includes questions regarding their socioeconomic status, access to medical services including health insurance services and their common source of information regarding antibiotics. Section 2 explored knowledge about antibiotics and Upper respiratory tract infections and their attitude towards using antibiotics for upper respiratory tract infections. Part 3 studied parents approach and expectations from pediatricians for prescribing antibiotics to their children suffering from URTIs. Also, this section explored their attitude towards using antibiotics without pediatrician advice and factors affecting this attitude.

#### Survey administration

We adopted a similar sampling strategy as conducted by SG Panagakou et al. in Greece as per the feasibility in the Makkah region [24]. The sample of the study contained parents from all geographical areas of the Holy Makkah region. The Kindergarten and elementary level schools were selected from various parts of Makkah city. A school-based stratified geographical cluster sampling technique was used to select a representative sample of students Kindergarten (5 years) and first-year students (6 years), whose parents were asked to fill in the questionnaire, by explaining the importance of study objectives and their contribution to the project. The questioner was distributed to each class by the class teachers in collaboration with the research team. The 1ST reminder to all nonrespondents was issued two weeks after the initial notification followed by two reminders at three weeks’ interval. Permission for survey administration was obtained by Schools directors based on ethical approval from Institutional Review Board of College of Pharmacy, Umm Al Qura University, Ministry of Education (Reference # UQU-COP-EA#143701). Stratification was obtained by selecting four main regions of the Makkah city to get representative samples.

### Sample size

The sample size for the current study was calculated using the online sample size calculator RaoSoft®. The minimum effective sample for this study was *n* = 558 with a confidence interval of 99%, response rate 30% and total estimated Makkah population of 5,979,719. However, upon the announcement of the survey, the number of parents who agreed to participate in this study was 650, of whom *n* = 570 completed the questionnaire and were considered for further analysis.

### Statistical analysis

All data were analysed using SPSS version 21®. Both descriptive and inferential statistics were applied to assess the correlated association with the self-directed use of antibiotics. Regression analysis was used to identify the factors having a significant association with the patient’s attitudes towards the use of antibiotics. *P*-values of less than 0.05 were considered statistically significant.

Linear regression uses the general linear eq. Y = b0 + ∑(biXi) + ϵY = b0 + ∑(biXi) + ϵ where YY is a continuous dependent variable and independent variables XiXi are *usually* continuous (but can also be binary, e.g. when the linear model is used in a t-test) or other discrete domains. ϵϵ is a term for the variance that is not explained by the model and is usually just called “error”. Individual dependent values denoted by YjYj can be solved by modifying the equation a little: Yj = b0 + ∑(biXij) + ϵj.

## Result

In this survey, five hundred and seventy parents completed the questionnaire. Approximately half of the parents had completed their college-level education while 73.3% had a moderate family income. The majority of the respondents (97.7%) were residents of Makkah and were living in a big town. More than 50% had 1 or 2 children, and only 5.6% reported a single-parent status. Fifty-three percent of the parents agreed that their children usually suffer from upper respiratory tract infections and most of the parents (86.1%) have no family or friendship relation with their pediatricians. Majority of the participants (86%) in this study reported that they had professional relationship with their pediatricians. Furthermore, 68% of the participants considered prescribers as the primary source of information about the judicious use of antibiotics (Table 1).

**Table 1:** Parents’ demographic characteristics (*N* = 570)

Variables	*n* (%)
Gender	
*Male*	42 (7.4)
*Female*	528 (92.6)
Age	
*20–30 years*	431 (75.6)
*31–40 years*	92 (16.1)
*41–60 years*	47 (8.2)
Educational status	
*Primary school*	2 (0.4)
*Secondary school*	4 (0.7)
*High school*	17 (3.0)
*College*	318 (55.8)
*University*	23 (4.0)
*No education*	206 (36.1)
Family income level	
*Very high*	18 (3.2)
*High*	111 (19.5)
*Moderate*	418 (73.3)
*Low*	14 (2.5)
*Very low*	9 (1.6)
Residence	
*Big town*	557 (97.7)
*Small town*	12 (2.1)
*Village*	1 (0.2)
Number of children	
*0*	72 (12.6)
*1*	217 (38.1)
*2*	126 (22.1)
*3*	67 (11.8)
*4*	52 (9.1)
*5*	18 (3.2)
*More than 5*	18 (3.2)
Parent of single child	
*Yes*	32 (5.6)
*No*	538 (94.4)
Do your children often suffer from Upper Respiratory Tract Infections?	
*Yes*	302 (53.0)
*No*	268 (47.0)
Sources of information you have about judicious antibiotic use	
*Physician*	386 (67.7)
*Television*	31 (5.4)
*Radio*	10 (1.8)
*Newspaper*	11 (1.9)
*Friend*	25 (4.4)
*Family relative*	48 (8.4)
*Other*	59 (10.4)

Parents’ knowledge about commonly used drugs in respiratory tract infections are shown in (Table 2); the response of the parents when they were asked to distinguish the antibiotics from a list of medicines including antibiotics, antipyretics, analgesics, mucolytics, antitussives, and bronchodilators. Most of them gave incorrect answers. Most of them had knowledge about OTC medications, but they were unable to identify antibiotics. While comparing males and female’s knowledge level, both groups were unaware of antibiotics given in list to identify except few males have identified Amoxil (Amoxicillin). Parents of age 20–30 years have good knowledge about the antibiotics and statistically significant for Augmentin (Co-amoxiclav), Ceclor (cefaclor) and Erythrocin (Erythromycin). Parents who are living in Makah have good knowledge and statistically significant for Erythrocin (Erythromycin). The detail response is shown in Table 2.

**Table 2:** Parents’ knowledge about commonly used drugs in respiratory tract infections

*Variables*		Percentage correct knowledge							
		Augmentin (Co-amoxiclav)	Areolin (salbutamol)	Depon (Paracetamol)	Ceclor (cefaclor)	Ponstan (mefenamic acid)	Amoxil (Amoxacillin)	Mucosolvan (Ambroxol HCL)	Erythrocin (Erythromycin)
Gender	*Male*	54.8%	90.5	95.2%	7.1	95.2	50*	95.2	16.7
	*Female*	47.7%	95.5	94.1%	6.8	93.8	29.7	94.5	7.4
Age (years)	*20–30*	43.2**	94.4	93.3	5.3*	93.5	27.4**	95.6	81
	*31–40*	68.5	98.9	97.8	14.1	96.7	44.6	92.4	7.6
	*41–60*	55.3	93.6	95.7	6.4	91.5	40.4	89.4	8.5
Parent of single child	*Yes*	31.3*	100	87.5	18.8*	100	25	90.6	18.8*
	*No*	49.3	94.8	94.6	6.1	93.5	31.6	94.8	7.4
Live in Makkah	*Yes*	47.7	95.4	94	7.1	93.3	31.2	94.2	6.9**
	*No*	55.1	91.8	95.9	4.1	100	32.7	98	20.4
Total *n* (%)		275 (48.2)	537 (94.2)	542 (95.1)	39 (6.8)	535 (93.9)	178 (31.2)	539 (94.6)	46 (8.1)

Pearson Chi-square; * *p* < 0.05;** *p* < 0.001

Understanding of antibiotics among respondent’s gender, age, living in Makkah and single parent status were assessed by applying a linear logistic regression model. Significant findings are obtained in a single child parent group (Table 3.1, 3.2).

**Table 3:** Understanding of antibiotics among respondent’s gender, age, living in Makkah and single parent status

Statement	Very much *N* (%)	Plenty *N* (%)	Not much *N* (%)	A little *N* (%)	Not at all *N* (%)	Gender OR (95% CI)	Age OR (95% CI)	Live in Makkah OR (95% CI)	Single Parent OR (95% CI)
*How much do you think that you are informed about judicious antibiotic use?*	27 (4.7)	107 (18.8)	280 (49.1)	94 (16.5)	36 (6.3)	− 0.013 (− 0.0405–0.299)	− 0.98* (− 0.315- -0.23)	− 0.035 (− 0.447–0.182)	0.086* (0.016–0.793)
*How many antibiotics do you think your child receives compared to other children?*	11 (1.9)	67 (11.8)	210 (36.8)	186 (32.6)	61 (10.7)	0.05 (− 0.154–0.603)	− 0.053 (− 0.258–0.058)	− 0.025 (− 0.441–0.236)	0.113* (0.115–0.991)
*How much do you pay attention to the possible side-effects of antibiotics?*	110 (19.3)	164 (28.8)	129 (22.6)	88 (15.4)	51 (8.9)	0.034 (− 0.256–0.605)	− 0.029 (− 0.243–0.118)	− 0.016 (− 0.464–0.315)	0.098* (0.090–1.052)
*Do you agree that you will be dissatisfied if your pediatrician does not prescribe an antibiotic for your child’s Upper Respiratory Tract Infection?*	52 (9.1)	98 (17.2)	183 (32.1)	139 (24.4)	68 (11.9)	0.011 (− 0.365–0.474)	− 0.015 (− 0.207–0.145)	− 0.026 (− 0.500–0.259)	0.078 (− 0.028–0.909)

Linear logistic regression, * = significant (*p* < 0.05); gender (ref male); age (20–30 years); live in Makkah (ref yes); Single parent (ref yes)

Linear logistic regression was applied from question no 16 to question 21. Q16 A: *Antibiotic must be administered in any case, once a child has a fever?* Have no significance with gender and education, however; age has statistical significance with OR = − 0.115 and CI 95% [− 0.426─ -0.061]. Q16 B: *As most of the Upper Respiratory Infections (like colds, flu, sore throats, ear infection) are of viral cause, they must not be cured with antibiotics?* Have no significance in gender and education, however; age has statistical significance with OR = − 0.137 and CI 95% [− 0.457─ -0.104]. Q16 E: *Antibiotics do not have side - effects?* Have significant association in gender with OR = 0.115 and CI 95% [0.150─ 0.950]*.* Q16 G*: Antibiotics decrease the complications of an Upper Respiratory Tract Infection?* have statistical significance with age OR = − 0.152 and CI 95% [− 0.411─ -0.115] and education OR = − 0.085 and CI 95% [− 0.093─ 0.000]. Q21B: *Would you change your pediatrician because in your opinion he/she does not prescribe antibiotics often enough for your child?* have statistical significance with age OR = 0.092 and CI 95% [− 0.041─ 0.914]; detailed are in Table 4.

**Table 4:** Relationship between standard coefficient beta and gender, age and education for Question 16 and 21 (N = 570)

Statement	SA *N*(%)	A *N*(%)	Uncertain *N*(%)	D *N* (%)	SD *N*(%)	Gender OR (95% CI)	Age OR (95% CI)	Education OR (95% CI)
Q16A	29 (5.1)	99 (17.1)	99 (17.1)	197 (34.6)	128 (22%)	−.035 (− 0.599, 0.245)	−0.115 (− 0.426, − 0.061) *	−0.062 (− 0.099, 0.015)
*Q16B*	45 (7.9)	151 (26.5)	139 (24.4)	156 (27.4)	53 (9.3)	0.05 (−0.163, 0.652)	−0.137 (− 0.457, − 0.104) *	−0.008 (− 0.061, 0.050)
*Q16C*	83 (14.6)	222 (38.9)	119 (20.9)	103 (18.1)	35 (6.1)	−0.004 (− 0.394, 0.355)	−0.054 (− 0.263, 0.062)	−0.055 (− 0.084, 0.018)
*Q16D*	61 (10.7)	201 (35.3)	205 (36)	67 (11.8)	26 (4.6)	0.024 (−0.238, 0.430)	0.009 (−0.129, 0.160)	− 0.054 (− 0.74, 0.016)
*Q16E*	26 (4.6)	49 (8.6)	124 (21.8)	202 (35.4)	153 (26.8)	0.115 (0.150, 0.950) *	0.046 (−0.081, 0.266)	−0.011 (− 0.061, 0.047)
*Q16F*	133 (23.3)	143 (25.1)	143 (26.8)	86 (15.1)	42 (7.4)	−0.022 (− 0.508, 0.297)	−0.169 (− 0.519, − 0.169)	−0.011 (− 0.055, 0.054)
*Q16G*	68 (11.9)	181 (31.8)	214 (37.5)	66 (11.8)	28 (4.9)	−0.026 (− 0.450, 0.234)	−0.152 (− 0.411, − 0.115) *	−0.085 (− 0.093, 0.000) *
*21A*	166 (29.1)	202 (35.4)	72 (12.6)	69 (12.1)	41 (7.2)	−0.066 (− 0.086, 0.734)	−0.079 (− 0.0341, 0.014)	−0.08 (− 0.108, 0.003)
*21B*	43 (7.5)	93 (16.3)	124 (21.8)	120 (29.8)	112 (19.6)	0.092 (0.041, 0.914) *	−0.053 (− 0.305, 0.072)	−0.052 (− 0.095, 0.023)
*21C*	59 (10.4)	164 (28.8)	116 (20.4)	88 (15.4)	123 (21.6)	0.013 (−0.386, 0.527)	−0.058 (− 0.331, 0.066)	−0.014 (− 0.072, 0.052)

Linear logistic regression, * = significant (*p* < 0.05); gender (ref male); age (20–30 years); Education (primary school)

*SA* Strongly Approve, *A* Approve, *N* Neutral, *D* Disapprove, *SD* Strongly Disapprove

Q16 A: Antibiotic must be administered in any case, once a child has fever?

Q16 B: As most of the Upper Respiratory Infections (like colds, flu, sore throats, ear infection) are of viral cause, they must not be cured with antibiotics?

Q16 C: If a child suffers from a flu or a cold, it will be cured more quickly if it is resistant bacteria?

Q16 D: Scientists can always produce new antibiotics that are able to kill the resistant bacteria?

Q16 E: Antibiotics do not have side - effects?

Q16 F: When antibiotics are administered when there is no special reason, their efficacy decreases and bacteria become more resistant?

Q16 G: Antibiotics decrease the complications of an Upper Respiratory Tract Infection?

Q21A: Do you believe antibiotics are used too much?

Q21B: Would you change your pediatrician because in your opinion he/she does not prescribe antibiotics often enough for your child?

Q21C: Would you change pediatrician because in your opinion he/she prescribe antibiotics for your child very often?

Q(A): If your pediatrician prescribes an antibiotic, how often do you ask him/her if it is actually necessary?

Q(B): How often do you praise a pediatrician if he/she prefers not to prescribe antibiotics?

Q(C): How often does your pediatrician recommend antibiotic therapy by phone?

Q(D): In case you strongly wish your child to receive antibiotics, how often do you directly ask your pediatrician for them?

Q(E): How often do you follow all your pediatrician’s instructions and advice?

Q(F): How often do you urge your pediatrician to prescribe antibiotic even when the diagnosis is not confirmed?

Q(G): How often does your pediatrician explain to you about your child’s condition and if they should or shouldn’t receive antibiotics?

Q(H): How often do you think that your pediatrician prescribes antibiotics only because you asked him/her?

**Fig. 1:**
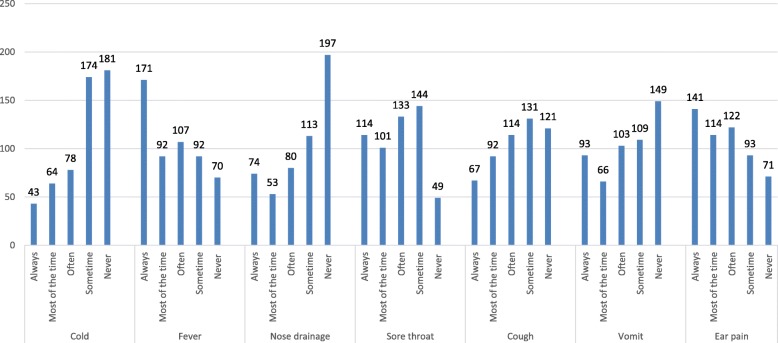
How often would you like your pediatricians to prescribe antibiotics for your child when it has a particular symptom?

**Fig. 2:**
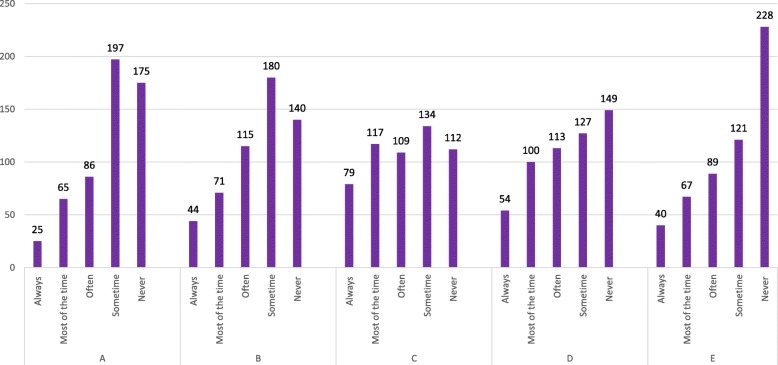
How often would you give your child antibiotics without a pediatrician’s advice, for the following reasons?

Figure 1; is related to question 19 How often would you like your pediatricians to prescribe antibiotics for your child when it has a particular symptom? Most of the time pediatricians prescribe antibiotics for fever, earache and sometimes for sore throats and coughs, but not usually for colds, nosebleeds and vomiting with responses on a scale from always, most of the time, often, some time and never. While Fig. 2 explains the reason why parents give their children antibiotics without a physician’s advice. Most parents would consider antibiotics for their children based on a previous prescription for similar symptoms. Sometimes self-administration of antibiotics occurred because of a lack of money or time and because the parents thought that symptoms were not severe enough to visit the pediatrician. A few parents reported that they gave antibiotics to their children on the recommendations of pharmacists, friends or relatives; with responses on a scale from always, most of the time, often, sometime and never. Figure 3.1 is related to parental practice towards antibiotic use in their children. Mostly parents questioned their pediatrician if an antibiotic prescription was necessary and they always followed the pediatrician’s advice. Parents report that the doctors often provide sufficient information regarding antibiotic use in their children. Most of the parents declared that they never received antibiotic recommendations from their pediatrician over the phone and most of them never insisted that their child’s doctor prescribed antibiotics when not recommended. Finally, very few parents believe that their pediatrician gives antibiotic prescriptions just because they asked them to do so.

**Fig. 3:**
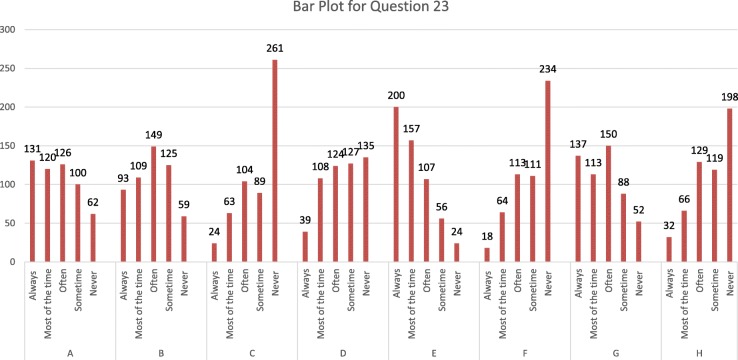
Parental practice towards antibiotic use in their children

## Discussion

Majority of the parents in the study expected antibiotics from their prescribers for the primary treatment of URTIs in their children. These findings suggest the need to educate parents about the effective and safe use of antibiotics in their children.

The percentage of parents demanding such inappropriate prescription for antibiotics reported in this study is almost twice the percentage of parents who had similar expectations for antibiotics in a previous study [27]. There is a common misconception that a specific treatment is available for every ailment, and antibiotics, in particular, are considered as miracle drugs that can cure everything from headaches to gastrointestinal diseases [28]. In two previous studies, such misconceptions held by parents about the effectiveness of antibiotics in treating viral URTIs have been attributed to inappropriate prescribing of antibiotics by physicians [20, 29]. In another study, 58% of the prescribers believed that their decision to prescribe antibiotics for a viral URTI such as common cold was influenced by parental pressure [18]. Parents’ lack of knowledge and awareness about the appropriate use of antibiotics, and the success they perceive about the effectiveness of antibiotics in the treatment of previous episodes of URTIs that were often self-limiting may explain the increase in the demand for antibiotics [30]. The incorrect perception of the general public about the effectiveness of antibiotics in treating viral URTIs has also been reported in a Dutch study where almost half of the participants wrongly recognised antibiotics to be useful in the treatment of viral infections [31].

Most of the participants in this study expressed their confidence in the advice provided to them by the prescribers. Some participants indicated that they would question their pediatrician about whether an antibiotic prescription was necessary, and stated that they always followed their pediatrician’s advice. Parents reported that they often received sufficient advice from their prescribers regarding antibiotic use in their children. These findings are similar to the findings of another study where around two-thirds of the participants considered prescribers to be the primary source of advice regarding the use of antibiotics [32]. However, a Chinese study indicated television to be the main source of information about antibiotics [33].

Participants were asked how many days they would allow before visiting their pediatrician, if their child presented with symptoms such as vomiting, cough, runny nose, sore throat and fever. More than half (65%) of the participants stated that they would visit a pediatrician within 1–2 days of their child developing any of the above symptoms, and 15% would contact their pediatrician on the same day. A Greek study that evaluated the knowledge, attitudes and practices of parents about antibiotic use for URTIs in children reported that Greek parents would visit pediatricians within two days of the development of symptoms [34].

Parents’ frequent visits to pediatricians, coupled with parental expectations to prescribe antibiotics, does not only result in the emergence of resistant strains of bacterial pathogens in the community but above all leads to an escalation in healthcare-related expenditure. It is believed that the majority of antibiotic prescriptions in pediatrics are issued for the treatment of virus-related URTIs [35]. Saudi Arabia, where antibiotics are available over the counter in pharmacies, presents an even bigger challenge to reduce the inappropriate use of antibiotics. As evident from the findings of the study, parents’ beliefs and their expectations of the prescribers determine the prescribing practice of antibiotics. The findings of this study, therefore, show the need to educate parents. Pharmacists, being some of the most accessible healthcare professionals, can play an important role in educating parents about the safe and effective use of antibiotics. Parents must be discouraged from seeking pediatricians’ advice at the onset of symptoms of virus-associated URTIs. Educating parents about the duration of URTIs and the often-self-limiting nature of such infections in children would help to allay the concerns of parents and would help in the reducing their dependency on antibiotics.

This study has some limitations. Participants were asked to self-report their understanding and awareness about antibiotics and experience of URTIs in their children. In the absence of any independent verification of information provided by the participants, their responses may not truly reflect their experience of URTIs and antibiotic use in their children. Furthermore, the questionnaire used in the study was in the English language, which may have presented a language barrier for some parents to understand and answer the questions correctly.

## Conclusions

Majority of Saudi parents have limited knowledge about antibiotics and URTIs and its management. Therefore, it is strongly recommended to educate parents about the safe and effective use of antibiotics. Provision of such education may assist in reducing the fears and concerns of parents about URTIs and thus may help in decreasing their dependency on antibiotics.

## Abbreviations

OTC: Over The Counter
URTIs: Upper Respiratory Tract Infections
